# Bioassay Guided Fractionation of *Senna singueana* and Its Potential for Development of Poultry Phytogenic Feed Additives

**DOI:** 10.3389/fvets.2021.800272

**Published:** 2022-01-13

**Authors:** Prosper Jambwa, Fikile N. Makhubu, Gift Matope, Gerda Fouche, Lyndy J. McGaw

**Affiliations:** ^1^Phytomedicine Programme, Department of Paraclinical Sciences, University of Pretoria, Onderstepoort, South Africa; ^2^Department of Veterinary Biosciences, University of Zimbabwe, Harare, Zimbabwe; ^3^Department of Veterinary Pathobiology, University of Zimbabwe, Harare, Zimbabwe; ^4^Department of Paraclinical Sciences, University of Pretoria, Onderstepoort, South Africa

**Keywords:** antibacterial, anti-lipoxygenase, antioxidant, cytotoxicity, phytogenic, poultry feed additives, *Senna singueana*

## Abstract

There has been burgeoning interest in plant-based feed additives following restrictions placed on the use of antibiotic feed additives in many countries. Phytogenic feed additives are recommended to have a range of useful properties to support the growth and development of poultry to a similar level as that obtained by supplementing feed with antibiotics. The aim of this study was to evaluate the antibacterial, anti-lipoxygenase and antioxidant activity, and *in vitro* safety of fractions and isolated compounds from leaves of *Senna singueana*. Antibacterial activities of the fractions and isolated compounds were determined against a panel of bacteria using a two-fold serial microdilution assay and qualitative bioautography assays. Anti-lipoxygenase activity was evaluated using the ferrous oxidation-xylenol orange (FOX) method. Antioxidant activity was assessed qualitatively and quantitatively using radical scavenging assays. Dichloromethane and ethyl acetate fractions from solvent-solvent partitioning had the best antibacterial activity with MIC values ranging from 156 to 313 μg/ml. Fractions obtained from column chromatography had significant to weak antibacterial activity with MIC values ranging from 50 to 1,250 μg/ml. Bioautography showed clear bands of bacterial inhibition, indicating the presence of a number of active compounds in several fractions. The ethyl acetate fraction and all the tested column fractions had potent anti-lipoxygenase activity with IC_50_ values of ≤2.5 μg/ml which were lower than that of quercetin (positive control), indicating anti-inflammatory potential. The ethyl acetate fraction and several column fractions had powerful antioxidant activity with IC_50_ values of ≤5 μg/ml in the ABTS assay. Cytotoxicity values against Vero kidney cells ranged from LC_50_ = 40.0–989.3 μg/ml. Bioassay-guided fractionation led to the isolation and identification of a known bioactive compound, luteolin. *S. singueana* is a promising candidate for the development of poultry phytogenic feed additives.

## Introduction

Phytogenic feed additives (PFAs) should have biological activity if they are to be used as alternatives to antibiotic growth promoters (AGPs). Reviews published to date have highlighted that phytogenic feed additives should have therapeutic value, such as antimicrobial, antioxidant, anti-inflammatory, immunostimulatory, anticoccidial, antiviral, and anti-ulcer (1–3). It therefore follows that compounds used in developing phytonutrient formulations for use as poultry growth promoters should preferably have multiple biological activity. They should therefore be isolated from plant parts rich in therapeutic phytochemicals.

*Senna singueana* (Delile) Lock belongs to the Caesalpiniaceae family and is native to tropical Africa, occurring throughout mainland tropical regions of Africa (4). Different parts of this plant species have numerous medicinal uses all over Africa. The plant is used to treat fever, malaria, pulmonary troubles, eye problems (conjunctivitis), skin disorders, venereal diseases, abdominal problems, bilharzia, impotence due to diabetes and wounds caused by leprosy, and syphilis (4, 5). It is also used as a purgative and as a lactation stimulant in both humans and animals (4, 5). In Zimbabwe, the leaves of *S. singueana* are used to treat a broad spectrum of poultry conditions such as coccidiosis, Newcastle disease, coughing, and flu-like symptoms (6).

Previous studies have shown that extracts of *S. singueana* leaves have moderate antibacterial activity against poultry pathogens, potent anti-lipoxygenase activity and powerful radical scavenging antioxidant activity (7). The bark methanol extract of *S. singueana* has also been reported to have remarkable hepatoprotective and anti-apoptotic properties (8), promoting further exploration of the plant for beneficial properties and potential uses. In view of its promising multiple biological activities, this study was designed to evaluate the antibacterial, anti-lipoxygenase, antioxidant and safety of *S. singueana* fractions and isolated compounds in order to assess the prospects of developing poultry PFAs from this plant species.

## Materials and Methods

### Plant Collection and Extraction

*Senna singueana* (Delile) Lock leaves were collected from Chipinge district (20° 23. 300′ S, 032 29. 691′), Manicaland Province in Zimbabwe. The plant was identified by Mr. Chapano, from the National Herbarium in Harare and authenticated by Ms. Magda Nel from the Department of Plant and Soil Science, University of Pretoria, South Africa. A voucher specimen was prepared and deposited in the H.G.W.J. Schweickerdt Herbarium (PRU 0125450) at the University of Pretoria, Pretoria, South Africa.

### Extraction and Solvent/Solvent Fractionation

The leaves were dried in a well-ventilated room at 25°C. Dried plant material was ground into a powder using a mill. Exhaustive extraction was carried out on powdered plant material (1,070.86 g) with 80% methanol to afford a crude extract (445.33 g). The crude extract (350.20 g) was subjected to solvent-solvent partitioning by dissolving in water (1,000 ml), and sequential partitioning with 1,000 ml each of *n*-hexane, dichloromethane, ethyl acetate and *n*-butanol. Each fraction was evaporated to dryness using a rotary evaporator (Büchi, Germany) under reduced pressure at 40°C.

### Column Chromatography

**Figure 1:**
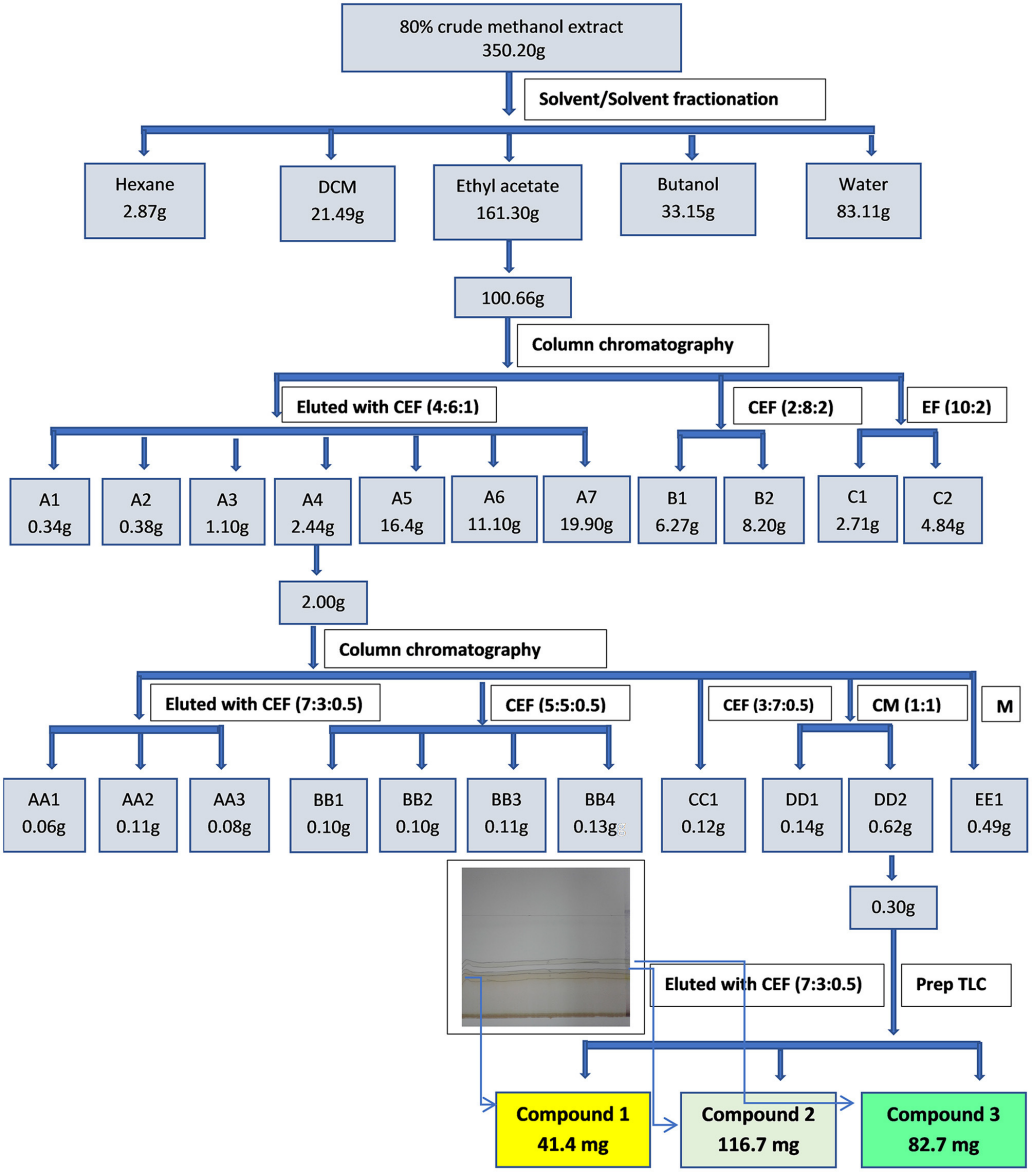
Extraction, fractionation and isolation of bioactive compounds from the leaf extract of *S. singueana*.

Part of the ethyl acetate fraction (100.66 g) was subjected to column chromatography. Silica gel (230–400 mesh, Merck) (1,063.18 g) was placed in a column with a diameter of 10 mm and an approximate height of 70 mm. The ethyl acetate fraction was loaded on the column and eluted with a combination of chloroform:ethyl acetate:formic acid (6:4:1) in increasing polarity as shown in Figure 1. A total of 194 fractions of ~50 ml each were obtained. The fractions were combined into eleven main fractions based on similarity of TLC phytochemical profiles. Fraction A4 was subjected to further column chromatography and was eluted with chloroform:ethyl acetate:formic acid (7:3:0.5) in increasing polarity (Figure 1) to afford eleven fractions based on TLC profiling. Sub-fraction DD2 (0.29 g) was subjected to preparative TLC using chloroform:ethyl acetate:formic acid (7:3:0.5) to yield compound 1 (yellow powder, 41.4 mg), compound 2 (116.7 mg) and compound 3 (82.7 mg).

### Thin Layer Chromatography Phytochemical Profiling

TLC phytochemical profiling was done by loading 10 μl of the crude extract, fraction or compound redissolved in their respective solvents or acetone to a concentration of 10 mg/ml on aluminium-backed silica gel plates (10 × 20 cm, 60 F254, Merck, United States). They were developed in three solvent systems of different polarities, namely BEA (benzene/ethanol/ammonium hydroxide (90:10:1)-non-polar solvent system, CEF (chloroform/ethyl acetate/formic acid (5:4:1)-intermediate polar solvent system, EMW (ethyl acetate/methanol/water (40:5.4:4)-polar solvent system (9). The separated phytochemicals were visualised under UV light at wavelengths of 254 nm and 365 nm and visible bands were marked. The TLC plates were then sprayed with freshly prepared vanillin-sulphuric acid reagent (0.1 g vanillin, 28 ml methanol, 1 ml sulphuric acid) and heated at 110°C until optimal colour development (10). Phytochemicals in fractions obtained from the column were analysed using the same procedure.

### Antibacterial Assay by TLC Bioautography

The compounds in the crude extract and fractions, as well as the purified compounds were developed as described above but the bioautograms were sprayed with respective cultures. After development, the TLC plates were dried overnight in a stream of cold air and sprayed with an actively growing concentrated suspension of strains of either *Staphylococcus aureus* (ATCC 29213)*, Salmonella enterica* subsp. *enterica* var. Enteritidis (*S. Enteritidis* ATCC 13076) or *Escherichia coli* (ATCC 25922). The plates were dried and incubated overnight at 37°C in closed, sterile, humidified plastic containers to allow growth of the bacteria on the plates. After incubation, the plates were sprayed with a sterile 2 mg/ml solution of *p*-iodonitrotetrazolium (INT, Sigma-Aldrich) salt and incubated for a further 1 h. The presence of clear zones on the chromatogram after the incubation period indicated inhibition of growth as the INT is metabolised to a coloured formazan product by the actively growing cells (11). The retention factors of the bands of inhibition were calculated.

### Qualitative Antioxidant Activity

TLC plates (10 × 20 cm, aluminium-backed, Merck, silica gel 60 F254) were loaded with 10 μl of the crude extract, fraction or compound (re-dissolved to 10 mg/ml) and dried before being developed in two mobile phase systems (CEF and EMW). To determine the antioxidant activity, the 1,1-diphenyl-2-picryl hydrazyl (DPPH) free radical test was performed directly by spraying the TLC plates with DPPH (0.2% w/v) in methanol to reveal the antioxidant activity of the fractions (12). A change of colour from the DPPH purple background to yellow indicated the presence of antioxidant compounds (13).

### Quantitative Antibacterial Assay by Minimum Inhibitory Concentration Assay

The antibacterial activity of the samples was determined by measuring the minimum inhibitory concentration (MIC) using a serial two-fold dilution method (14). The following test organisms were used: *Staphylococcus aureus* (ATCC 29213)*, E. coli* (ATCC 25922)*, S*. Enteritidis (ATCC 13076), and a clinical strain of *E. coli* obtained from the Department of Veterinary Tropical Diseases, University of Pretoria.

The four bacterial cultures were prepared by inoculating a single colony from an agar plate into 10 ml of sterilised Mueller-Hinton (MH) broth (Merck, South Africa) and incubating at 37°C in an MRC orbital shaker (150 rpm) incubator (United Scientific, South Africa) for 18 to 20 h prior to the experiment. Following incubation, each bacterial strain was diluted in MH broth (Merck, South Africa) and the absorbance was measured at a wavelength of 560 nm using a spectrophotometer (Epoch microplate reader: BioTek, United States). Absorbance was adjusted to match that of a McFarland standard No 1 (corresponding to ~3 × 10^8^ colony forming units per ml, cfu/ml).

The assay was performed in microtitre plates (Lasec, South Africa) by adding 100 μl of sterile water to all wells. In the first row, 100 μl of extract, fraction or compound were added in triplicate and serially diluted two-fold to the last well, from which 100 μl were then discarded. Gentamicin (Virbac, South Africa) was used as a positive control and a sterility control containing only water was included. This was followed by addition of 100 μl of the bacterial suspension to each well (except for the sterility control). The plates were sealed with parafilm and incubated at 37°C (IncoTherm, Labotec). After 24 h, 40 μl of a 0.2 mg/ml solution of INT was added to each well and the plate further incubated for at least half an hour to ensure adequate colour development. INT is a dehydrogenase activity detecting reagent, which is converted into an intensely coloured red-purple formazan by metabolically active micro-organisms. Inhibition of growth was indicated by a clear solution or a noticeable decrease in colour reaction. This value was taken as the MIC of the sample. The experiments were conducted twice.

### Cytotoxicity Evaluation

Cytotoxicity evaluation was done on fractions which showed good activity and the isolated compounds. The cytotoxic effect of the fractions and the isolated compounds was determined using an *in vitro* assay with Vero monkey kidney cells (15). The growth medium used was Minimal Essential Medium (MEM, Whitehead Scientific) supplemented with 0.1% gentamicin (Virbac) and 5% foetal calf serum (Highveld Biological). The cells were seeded at a density of 10 000 cells/per well in 96-well-microtitre plates. The plates were incubated at 37°C in a 5% CO_2_ incubator in a humidified environment for 24 h to allow cell attachment.

After incubation, the medium was aspirated and replaced with fresh MEM. The fractions/compounds (100 μl) of varying concentrations were added to the wells containing cells. The anticancer compound doxorubicin (Pfizer Laboratories) was used as a positive control. A suitable blank control with equivalent concentrations of fresh medium was also included and the plates were further incubated for 48 h in a CO_2_ incubator. Subsequently, the medium in each well was aspirated from the cells, which were washed with phosphate-buffered saline (PBS) and fresh medium was then added to each well. A 30 μl aliquot of MTT (5 mg/ml in PBS) was added to each well and the plates were incubated at 37°C for 4 h. The medium was then aspirated from wells and 50 μl DMSO was added to each well to solubilise the formed formazan crystals. The absorbance was measured on a BioTek Synergy microtitre plate reader at 570 nm. Cell growth inhibition for each extract was expressed in terms of LC_50_ values. The selectivity index (SI) was also calculated. The cytotoxicity assay was repeated thrice.

### Quantitative Determination of Antioxidant Activity

#### DPPH (1,1-Diphenyl-2-Picryl Hydrazyl) Free Radical Assay

The antioxidant activities of the samples were measured in terms of radical scavenging ability using the stable radical (DPPH) method of Brand-Williams et al. (12) with some modifications. Methanol solutions (40 μl) of the samples and positive controls (Trolox and ascorbic acid) at various concentrations (0.1–100 μg/ml) were prepared by serial dilution in a 96 well-microtitre plate. One hundred and sixty (160) μl of DPPH in methanol adjusted to an absorbance between 0.9 and 1.0 was added and the plates were incubated in the dark at room temperature (25°C) for 30 min. Absorbance was measured against a blank with a microtitre plate reader (Epoch, BioTek, United States) at 516 nm. The DPPH scavenging effect was determined using the following formula:


(1) $$\begin{array}{l} \text{DPPH Scavenging Effect}\left(\text{\%}\right)=\left[\left(\text{A}1-\text{A}2/\text{A}1\right)\right]\;\times \;100 \end{array}$$

Where A1 is the absorbance of the control reaction and A2 is the absorbance in the presence of the sample. Trolox and ascorbic acid were used as controls. The experiments were conducted twice.

#### ABTS [2,2-Azino-Bis (3-Ethylbenzothiazoline-6 Sulfonic Acid)] Free-Radical-Scavenging Assay

The free radical-scavenging activity as a measure of hydrogen donating capacity was determined by using the ABTS cation decolourization method of Re et al. (16) with some modifications. ABTS radical solution (7 μM) was prepared by dissolving 1.32 × 10^4^ μg of ABTS in 10 ml of 50% methanolic solution and 7.68 × 10^4^ μg of potassium persulphate (K_2_S_2_O_4_) in 10 ml of distilled water. The two solutions were mixed together and made up to 200 ml with 50% methanolic solution, and kept in the dark at room temperature, 25°C for 12 h. Prior to running the assay, the ABTS radical solution was diluted with 50% methanolic solution to an absorbance between 0.7 and 0.8 at 734 nm. The samples were serially diluted (40 μl) (0.1–100 μg/ml) in 96 well-microtitre plates and 160 μl of ABTS radical solution was added to each well. The absorbance readings were taken after exactly 6 min of reaction and blanks were prepared using the respective samples without ABTS radical. The scavenging effect was calculated using the following formula:


(2) $$\begin{array}{l} \text{ABTS Scavenging Effect}\left(\text{\%}\right)=\left[\left(\text{A}1-\text{A}2/\text{A}1\right)\right]\;\times \;100 \end{array}$$

The IC_50_ values were calculated from a graph plotted as inhibition percentage against the concentration. A Trolox standard curve was drawn by plotting percentage inhibition of the ABTS+ radical against the concentration of Trolox. Data from the test samples were analysed in a similar manner.

### Anti-Lipoxygenase (15-LOX) Assay

Lipoxygenase (LOX) activity of the samples was determined spectrophotometrically according to published methods (17, 18). LOX inhibition was determined spectrophotometrically based on the formation of the complex Fe3+/xylenol orange as described by Pinto et al. (19). Briefly, 20 μl of Tris-HCl buffer (pH 7.4) was added to all wells of the 96-well-microplates. This was followed by the addition of 20 μl of the fractions (1 or 0.5 mg/ml) in the first row of the plate which was serially diluted. Quercetin served as the positive control, and the buffer was used as a negative control. After the serial dilution, 40 μl of the lipoxygenase enzyme (Sigma Aldrich, Germany) was added to each well and the plates were incubated at room temperature 25°C for 5 min. After incubation, 40 μl of linoleic acid (final concentration, 140 μM) prepared in Tris-HCl buffer (50 mM, pH 7.4) was added to the well (except for the blanks). The plates were incubated at 25°C for 20 min in the dark. After incubation, 100 μl of freshly prepared ferrous oxidation–xylenol orange (FOX) reagent [sulfuric acid (30 mM), xylenol orange (100 μM), iron (II) sulphate (100 μM) in methanol/water (9:1)] was added to all wells. The plates were further incubated at 25°C for 30 min in the dark, 40 μl of linoleic acid was then added to the blanks. The absorbance was measured at 560 nm. The selectivity index (SI) values regarding anti-LOX activity were calculated by dividing cytotoxicity LC_50_ values by the IC_50_ values of relevant bioactivity (SI = LC_50_/IC_50_) (20). The experiments were conducted twice.

### Structure Elucidation of Compounds

Structures of the isolated compounds were identified using nuclear magnetic resonance (NMR) (1D) spectroscopy. ^1^HNMR data was acquired on a 400 MHz NMR spectrometer (Bruker Avance III 400 MHz) while ^13^CNMR data was acquired on a 125 MHz NMR spectrometer. The structures of the isolated compound that was able to be identified was confirmed by comparison of the NMR data with those published previously. The molecular weight of the compound was confirmed using Ultra Performance Liquid Chromatography-Mass Spectrometry (UPLC-MS).

### Data Analysis

Data were presented as mean ± standard deviation (SD) of the determinations. The Shapiro-Wilk Normality Test was used to check for normality of antioxidant and anti-LOX data. The hypothesis of normality was rejected when the *p* ≤ 0.05. Log transformation of data was carried out on data which was not normally distributed. Statistical analyses of the antioxidant and anti-LOX data was then performed using the Tukey– Kramer multiple comparison *post-hoc* test following one way ANOVA. A *P* < 0.05 was considered statistically significant. The data were computed using IBM SPSS Statistics.

## Results

### Qualitative Antibacterial Activity

**Figure 2:**
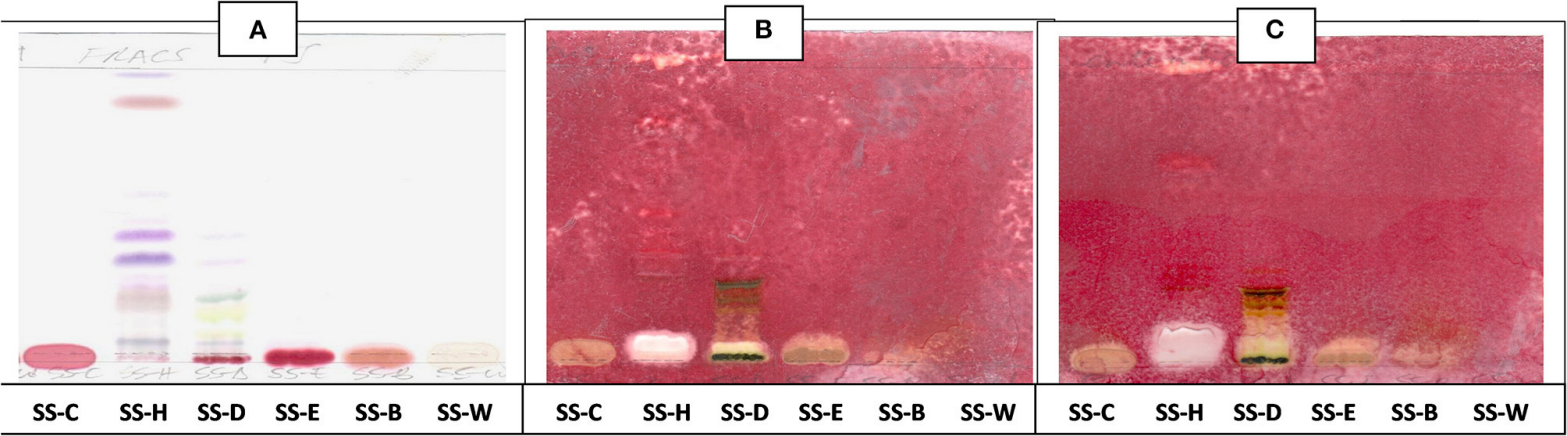
**(A)** Chromatogram developed in BEA [benzene: ethanol: ammonium hydroxide (90:10:1)] of *S. singueana* fractions sprayed with vanillin. **(B)** Bioautography of *S. aureus*–TLC plate developed with BEA (90:10:1). **(C)** Bioautography of *S. Enteritidis*–TLC plate developed with BEA (90:10:1). SS-C, *S. singueana* crude extract; SS-H, hexane fraction; SS-D, dichloromethane fraction; SS-E, ethyl acetate fraction; SS-B, butanol fraction; SS-W, Water fraction. White bands indicate compounds that inhibit bacteria.

**Figure 3:**
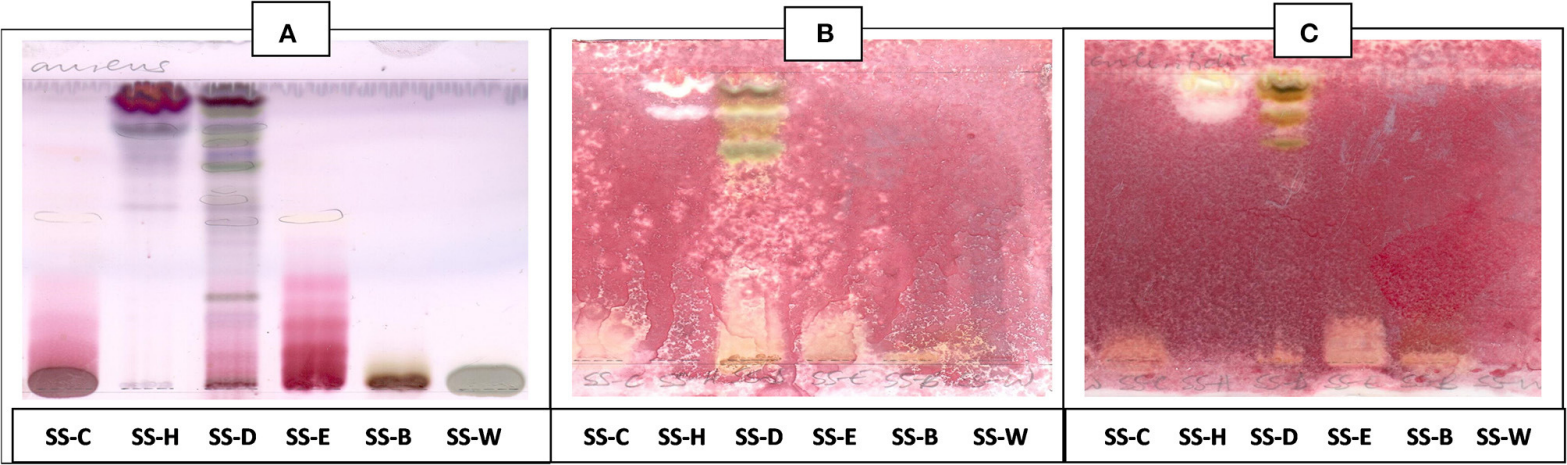
**(A)** Chromatogram developed in CEF [chloroform: ethyl acetate: formic acid (5:4:1)] of *S. singueana* fractions sprayed with vanillin. **(B)** Bioautography of *S. aureus*–TLC plate developed with CEF (5:4:1). **(C)** Bioautography of *S. Enteritidis*–TLC plate developed with CEF (5:4:1). SS-C, *S. singueana* crude extract; SS-H, hexane fraction; SS-D, dichloromethane fraction; SS-E, ethyl acetate fraction; SS-B, butanol fraction; SS-W, Water fraction. White bands indicate compounds that inhibit bacteria.

**Figure 4:**
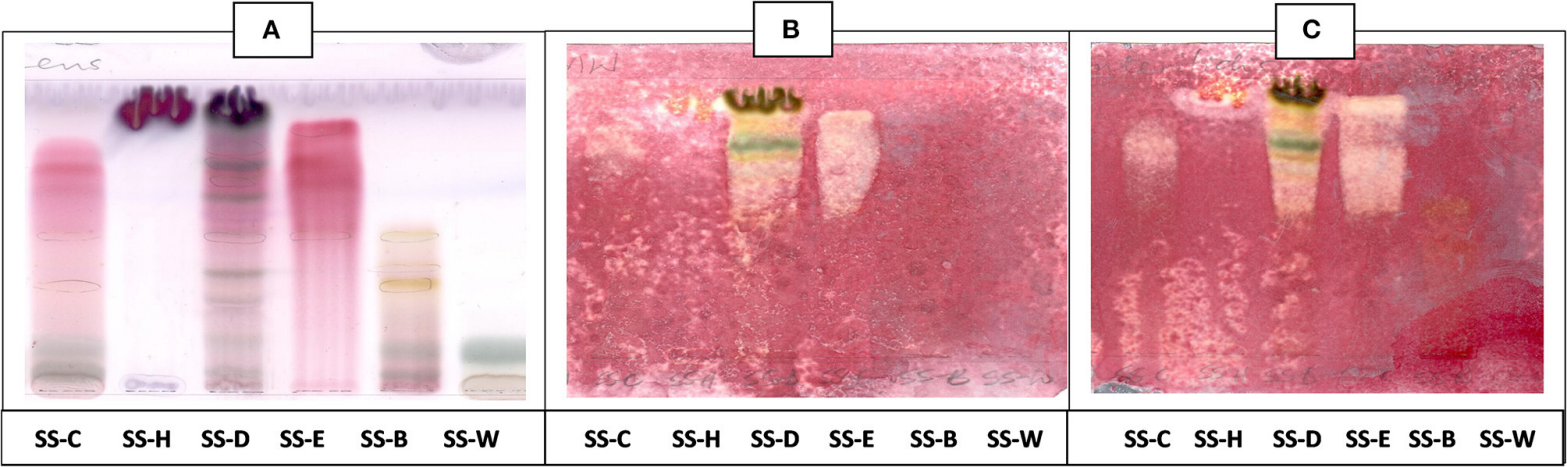
**(A)** Chromatogram developed in EMW [ethyl acetate: methanol: water (40:5.4:4)] of *S. singueana* fractions sprayed with vanillin. **(B)** Bioautography of *S. aureus*–TLC plate developed with EMW (40:5.4:4). **(C)** Bioautography of *S. Enteritidis*–TLC plate developed with EMW (40:5.4:4). SS-C, *S. singueana* crude extract; SS-H, hexane fraction; SS-D, dichloromethane fraction; SS-E, ethyl acetate fraction; SS-B, butanol fraction; SS-W, Water fraction. White bands indicate compounds that inhibit bacteria.

The antibacterial activity with bioautography method (Figures 2–4) indicated that the *n*-hexane, dichloromethane and ethyl acetate fractions had antibacterial activity against *S. aureus* and *S*. Enteritidis (Figures 2B,C) with the *n-*hexane fraction having prominent bands when using BEA as mobile phase. The antibacterial compounds did not move from the point of origin.

**Figure 5:**
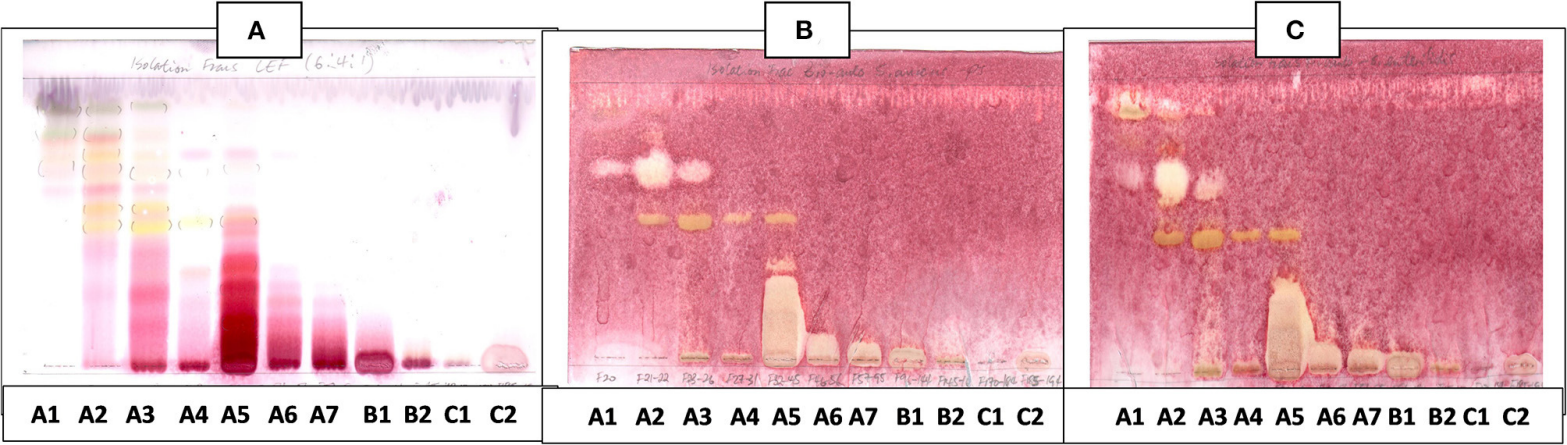
**(A)** Chromatogram developed in CEF [chloroform: ethyl acetate: formic acid (6:4:1)] of 11 combined fractions obtained from the first column sprayed with vanillin. **(B)** Bioautography of *S. aureus* of the 11 combined column fractions–TLC plate developed with CEF (6:4:1). **(C)** Bioautography of *S. Enteritidis* of the 11 combined column fractions–TLC plate developed with CEF (6:4:1). Yellow and white bands indicate compounds that inhibit bacteria.

**Figure 6:**
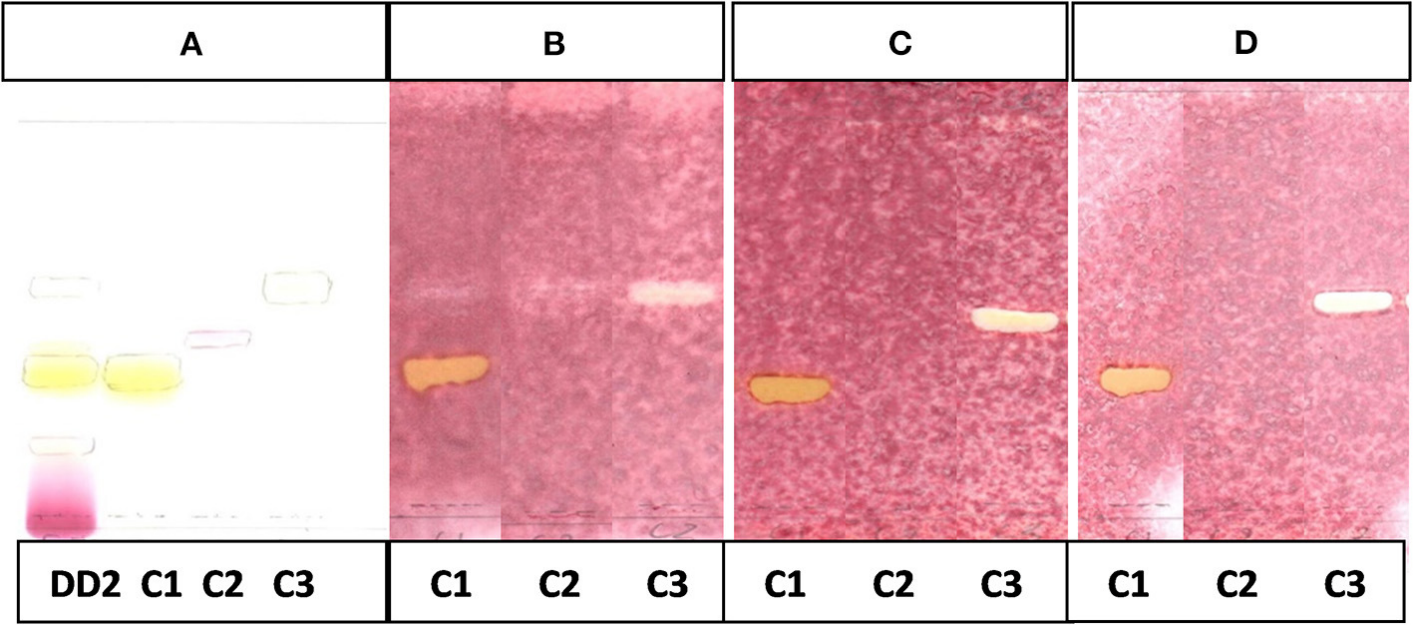
**(A)** Chromatogram developed in CEF (7:3:0.5) of sub fraction DD2 and the 3 compounds sprayed with vanillin.**(B)** Bioautography of *S. aureus* of the 3 compounds. TLC developed in CEF (7:3:0.5). **(C)** Bioautography of *E. coli* of the 3 compounds. TLC developed in CEF (7:3:0.5). **(D)** Bioautography of *S. Enteritidis* of the 3 compounds. TLC developed in CEF (7:3:0.5). **(B–D)** Composite images of TLC plates run separately for each compound. C1, compound 1; C2, compound 2; C3, compound 3.

The CEF and EMW mobile phase separated antibacterial compounds in the n-hexane, dichloromethane and ethyl acetate fractions, with the dichloromethane and ethyl acetate fractions having significant bands of inhibition (Figures 3B,C, 4B,C). However, the active compounds of the ethyl acetate fraction did not separate into clear bands. Fractions obtained from the column, namely A1, A2, A3, A4, A5, A6, A7, and B1 had active compounds, with the A1, A2, A3, A4 fractions showing better separation of active bands (Figures 5B,C). Bioautography of the isolated compounds showed that compound 1 (R_f_ value = 0.32) and compound 3 (R_f_ value = 0.51) were active against *S. aureus, E. coli* and *S. Enteritidis* (Figures 6B–D).

### Qualitative Antioxidant Activity

**Figure 7:**
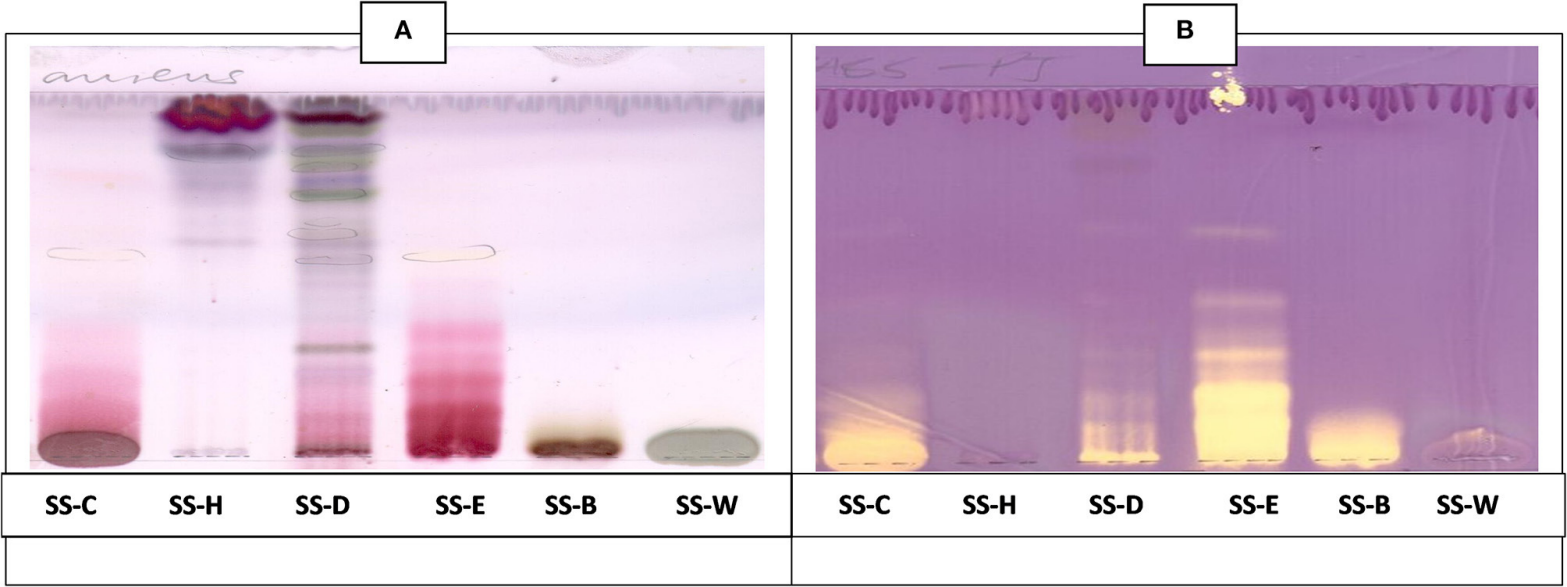
**(A)** Chromatogram developed in CEF [chloroform: ethyl acetate: formic acid (5:4:1)] of *S. singueana* fractions sprayed with vanillin. **(B)** Antioxidant bioautography–TLC plate developed with CEF (5:4:1) and sprayed with DPPH. Yellowish bands indicate compounds antioxidant activity. SS-C, *S. singueana* crude extract; SS-H, hexane fraction; SS-D, dichloromethane fraction; SS-E, ethyl acetate fraction; SS-B, butanol fraction; SS-W, Water fraction.

**Figure 8:**
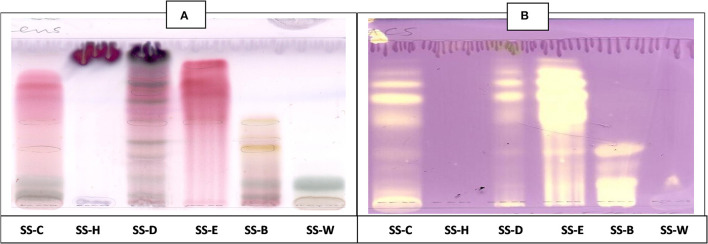
**(A)** Chromatogram developed in EMW [ethyl acetate: methanol: water (40:5.4:4)] of *S. singueana* fractions sprayed with vanillin. **(B)** Antioxidant bioautography–TLC plate developed with EMW (40:5.4:4) and sprayed with DPPH. SS-C, *S. singueana* crude extract; SS-H, hexane fraction; SS-D, dichloromethane fraction; SS-E, ethyl acetate fraction; SS-B, butanol fraction; SS-W, Water.

**Figure 9:**
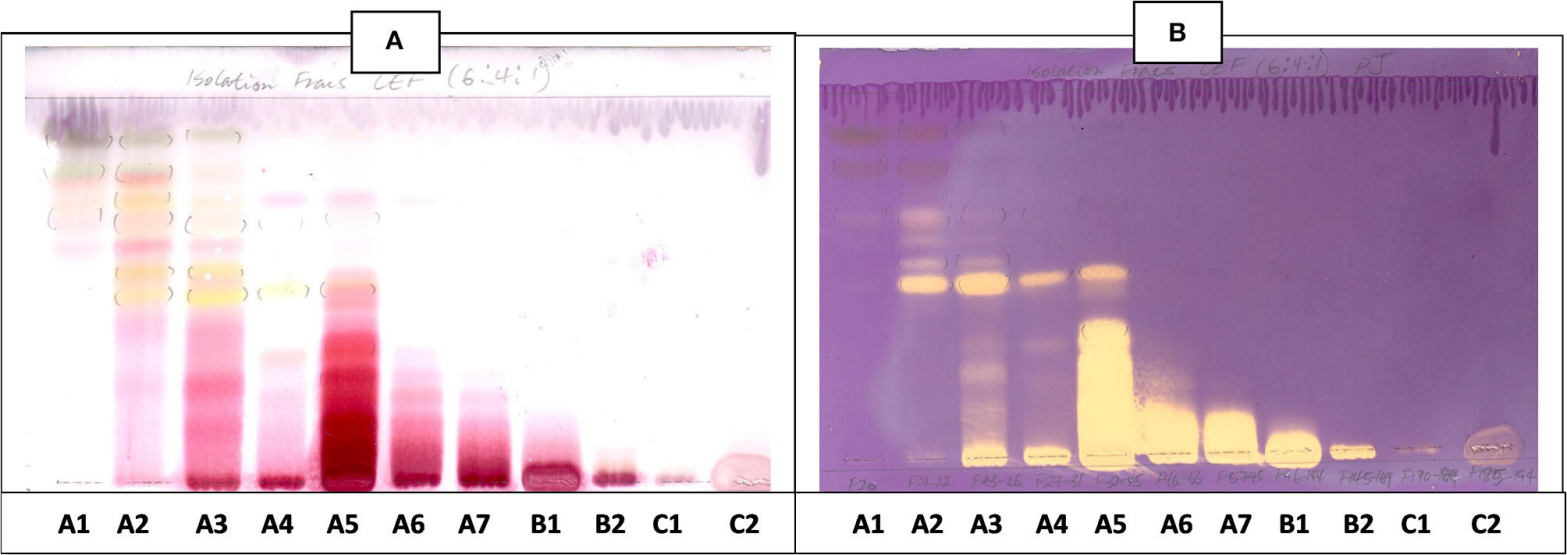
**(A)** Chromatogram developed in CEF [chloroform: ethyl acetate: formic acid (6:4:1)] of 11 combined fractions obtained from the first column sprayed with vanillin. **(B)** Antioxidant bioautography–TLC plate developed with CEF (6:4:1) and sprayed with DPPH. Yellowish bands indicate compounds antioxidant activity.

**Figure 10:**
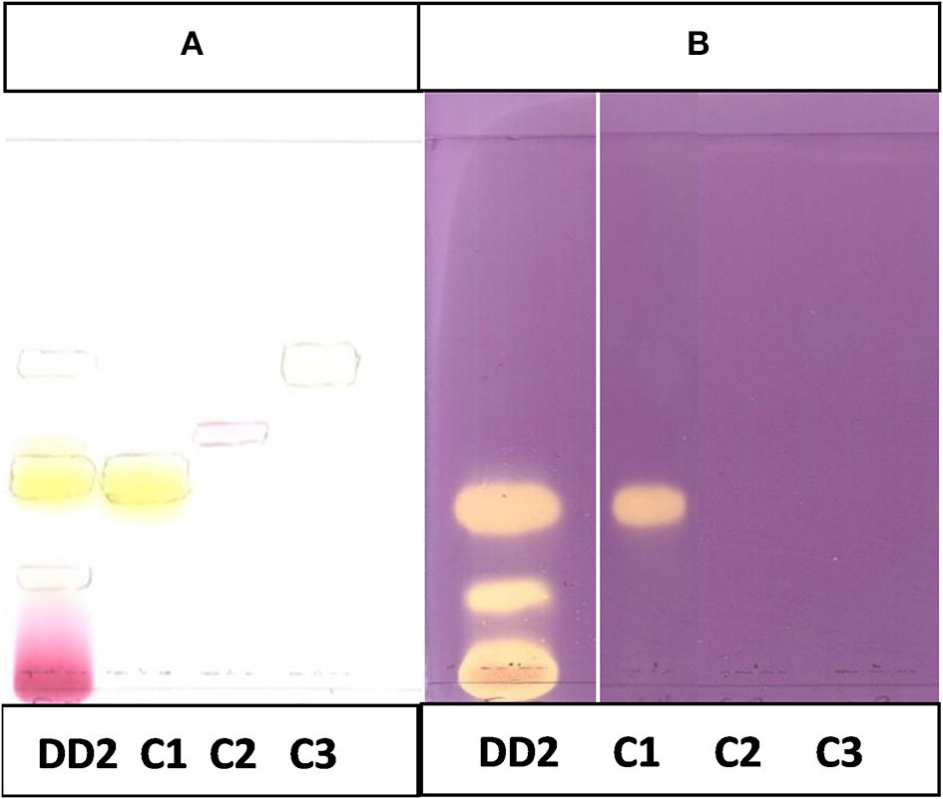
**(A)** Chromatogram developed in CEF (7:3:0.5) of sub fraction DD2 and the 3 compounds sprayed with vanillin. **(B)** Chromatogram developed in CEF (7:3:0.5) of sub fraction DD2 and the 3 compounds sprayed with DPPH.

The CEF and EMW antioxidant bioautography (Figures 7, 8) showed that the dichloromethane, ethyl acetate and the butanol fraction had antioxidant activity with the ethyl acetate profile showing prominent bands of DPPH bleaching (Figures 7B, 8B). The antioxidant compounds of the ethyl acetate fractions did not separate into distinct bands. Antioxidant bioautography also showed that column fractions A2, A3, A4, A5, A6, A7, B1, and B2 had bands of antioxidant activity (Figure 9B). Compound 1 also had antioxidant activity in the bioautography assay (Figure 10B).

### Quantitative Antibacterial Activity

MIC values ≤ 100 μg/ml indicate significant activity, 100 < MIC ≤ 625 μg/ml moderate activity and values >625 μg/ml indicate weak activity (21). Of the fractions obtained from solvent/solvent-solvent partitioning solvent fractionation, the dichloromethane and ethyl acetate fractions had the best antibacterial activity with MIC values ranging from 156 to 313 μg/ml against the three ATCC strains and *E. coli* clinical strain (Table 1). These two fractions had moderate antibacterial activity against the tested strains. The fractions obtained from the first column had significant to weak antibacterial activity against the bacterial strains with MIC values ranging from 50 to 1,250 μg/ml. Fraction A2, A3, A4, A6, A7, and B1 had significant antibacterial activity against the *E. coli* ATCC strain while only fractions A3 and A6 had noteworthy activity against the *S. Enteritidis* ATCC strain. Fraction A3, A4, and A6 also had significant activity against the *E. coli* clinical strain. None of the fractions had significant activity against the *S. aureus* ATCC strain, with most having moderate activity against this strain. The isolated compound, luteolin had relatively weak antibacterial activity against all the tested bacterial strains.

**Table 1:** Yield and minimum inhibitory concentrations (MICs), and of *S. singeuana* fractions against ATCC strains.

**Sample**	**%yield**	***Staphylococcus aureus* (ATCC 29213)**	***Escherichia coli* (ATCC 25922)**	***Salmonella Enteritidis* (ATCC 13076)**	***Escherichia coli* (clinical strain)**
		**MIC (μg/ml)**	**MIC (μg/ml)**	**MIC (μg/ml)**	**MIC (μg/ml)**
Crude extract	41.59	625 ± 0.00	235 ± 86	625 ± 0.00	469 ± 170
Hexane frac	0.82	2,500 ± 0.00	2,500 ± 0.00	1.25 ± 0.00	469 ± 170
Dichloromethane	6.14	313 ± 0.00	156 ± 0.00	313 ± 0.00	104 ± 40
Ethyl acetate frac	46.05	235 ± 86	156 ± 0.00	313 ± 0.00	117 ± 43
Butanol frac	9.47	938 ± 342	156 ± 0.00	625 ± 0.00	469 ± 170
Water frac	23.73	>2,500	>2,500	>2,500	>2,500
A1	0.34	938 ± 313	313 ± 0.00	729 ± 56	938 ± 342
A2	0.38	156 ± 0.00	**78** **±** **0.00**	117 ± 42	156 ± 0.00
A3	1.09	156 ± 0.00	**52** **±** **20**	**78** **±** **0.00**	**78** **±** **0.00**
A4	2.42	117 ± 39	**78** **±** **0.00**	156 ± 0.00	**78** **±** **0.00**
A5	16.29	235 ± 78	156 ± 0.00	313 ± 0.00	313 ± 0.00
A6	11.03	156 ± 0.00	**65** **±** **20**	**78** **±** **0.00**	**78** **±** **0.00**
A7	19.77	156 ± 0.00	**78** **±** **0.00**	156 ± 0.00	156 ± 0.00
B1	6.23	156 ± 0.00	**78** **±** **0.00**	156 ± 0.00	156 ± 0.00
B2	8.15	313 ± 0.00	156 ± 0.00	313 ± 0.00	235 ± 86.00
C1	2.69	1,042 ± 295	938 ± 342	938 ± 0.342	625 ± 0.00
C2	4.81	1,250 ± 0.00	938 ± 342	1,250 ± 0.00	1,250 ± 0.00
Compound 1	13.80	625 ± 0.00	156 ± 0.00	1 250 ± 0.00	156 ± 0.00
Compound 2	38.90	313 ± 0.00	>2,500	>2,500	>2,500
Compound 3	27.57	313 ± 0.00	625 ± 0.00	>2,500	625 ± 0.00
Gentamicin	N/A	20.00 ± 0.00	20.00 ± 0.00	20.00 ± 0.00	20.00 ± 0.00

*Frac, fraction, A1–C2 indicates column fractions. MIC values ≤ 100 μg/ml indicate significant activity, 100 < MIC ≤ 625 μg/ml moderate activity and values >625 μg/ml indicate weak activity. Values in bold indicate MICs lower than 100 μg/mL*.

### Cytotoxicity Results

According to the National Cancer Institute, there are four group classifications for cytotoxicity evaluation: Very active (LC_50_ ≤ 20 μg/ml), moderately active (LC_50_ > 20–100 μg/ml), weakly active (LC_50_ > 100–1,000 μg/ml), and inactive (LC_50_ > 1,000 μg/ml) (22, 23). The ethyl acetate fraction, fractions 3 and A6, compounds 1 and 3 had moderate cytotoxicity against the Vero monkey cells whilst the dichloromethane fraction, Fraction A4, A5, A6, B1, and compound 2 exhibited weak toxicity with LC_50_ values of >100 (Table 2).

**Table 2:** Cytotoxicity (LC_50_ values) and selective index of the *S. singueana* fractions with respect to antibacterial activity.

**Fraction**	**LC_**50**_**	**Test organisms and Selectivity index (SI)** **=** **LC**_**50**_**/MIC**			
	**(μg/ml)**	***S. aureus* (ATCC 29213)**	***E. coli* (ATCC 25922)**	***S. Enteritidis* (ATCC 13076)**	***E. coli* (Clinical strain)**
Dichloromethane	40.0 ± 2.8	0.1	0.3	0.1	0.4
Ethyl acetate	139.3 ± 19.5	0.6	0.9	0.4	1.2
A2	63.6 ± 16.3	0.4	0.8	0.5	0.4
A3	85.2 ± 6.7	0.5	**1.6**	**1.1**	**1.1**
A4	151.5 ± 16.1	**1.3**	**1.9**	**1.0**	**1.9**
A5	989.3 ± 61.3	**4.2**	**6.3**	**3.2**	**3.2**
A6	109.6 ± 20.0	0.7	**1.7**	**1.4**	**1.4**
B1	142.2 ± 7.3	0.4	**1.8**	0.9	0.9
Compound 1	92.9 ± 1.7	0.1	0.6	0.6	0.6
Compound 2	309.5 ± 72.8	1.0	ND	ND	ND
Compound 3	79.4 ± 4.3	0.3	0.1	ND	0.1
Doxorubicin	9.00 ± 1.28	N/A	N/A	N/A	N/A

*Values in bold indicate SI > 1, ND, Not determined; N/A, Not applicable*.

### Quantitative Antioxidant Activity

The ethyl acetate fraction had powerful antioxidant activity with IC_50_ values of 2.69 and 2.46 μg/ml in the DPPH and ABTS assays, respectively (Table 3). Fractions A3, A4, A5, A6, A7, and B1 also showed remarkable antioxidant activity with IC_50_ values of <2.5 μg/ml in the ABTS assay. Fraction A4 had the best antioxidant activity in the DPPH assay with an IC_50_ value of 3.05 μg/ml.

**Table 3:** Antioxidant activity of *S. singueana* fractions.

**Fraction**	**DPPH IC_**50**_ (μg/ml)**	**ABTS IC_**50**_ (μg/ml)**
Crude extract	6.08 ± 0.33^a^	1.82 ± 0.77 (1.80)^a^
Hexane	158.79 ± 31.20^d^	126.30 ± 9.76^c^
Dichloromethane	9.33 ± 1.17^a^	4.06 ± 1.17^a^
Ethyl acetate	2.69 ± 0.22^a^	2.46 ± 0.17^a^
Butanol	9.85 ± 0.94^a^	3.98 ± 0.62^a^
Water	274.41 ± 6.26^e^	53.84 ± 22.64^b^
A1	108.42 ± 12.52^c^	60.09 ± 9.18^b^
A2	8.86 ± 1.10^a^	7.07 ± 0.87^a^
A3	3.36 ± 0.29 (4.44)^a^	1.88 ± 0.26 (2.37)^a^
A4	3.05 ± 0.03 (3.38)^a^	1.30 ± 0.12 (1.78)^a^
A5	6.29 ± 0.67^a^	2.16 ± 0.48 (2.60)^a^
A6	7.50 ± 0.40 (6.77)^a^	1.75 ± 0.24 (2.22)^a^
A7	6.64 ± 0.12 (6.14)^a^	1.64 ± 0.23 (2.11)^a^
B1	6.25 ± 0.51 (5.92)^a^	1.96 ± 0.23 (2.43)^a^
B2	7.63 ± 1.51 (7.09)^a^	2.55 ± 0.32 (3.02)^a^
C1	12.55 ± 1.05^a^	10.18 ± 3.51^a^
C2	60.65 ± 7.23^b^	199.79 ± 50.00^d^
Luteolin (Compound 1)	5.92 ± 0.64^a^	8.17 ± 0.80^a^
Compound 2	>100	57.40 ± 1.52^b^
Compound 3	>100	>100
Ascorbic acid	1.97 ± 0.21 (2.45)^a^	1.90 ± 0.07 (2.45)^a^
Trolox	3.19 ± 0.32 (4.02)^a^	2.21 ± 0.30 (2.76)^a^

*N = 3, mean values within a column with different superscript letters are significantly different at p < 0.05. The values in brackets indicate IC_50_ values obtained after log transformation of data which was not normally distributed*.

### Anti-Lipoxygenase Activity

The ethyl acetate fraction and all five fractions from the first column which were tested had potent anti-lipoxygenase activity with each having IC_50_ values of <2.5 μg/ml (Table 4). Similar to the antioxidant results, fraction A4 had the most potent anti-lipoxygenase activity with an IC_50_ value of 0.32 μg/ml. The selective index (SI) values regarding anti-lipoxygenase activity (20) of the dichloromethane, ethyl acetate and the five column fractions (A3, A4, A5, A6, B1) were >10.

**Table 4:** Anti-lipoxygenase activity of *S. singueana* fractions.

**Fraction**	**15-Lox IC_**50**_ (μg/ml)**	**LC_**50**_ (μg/ml)**	**Selective index**
Dichloromethane	5.15 ± 0.07^c^	40.0 ± 2.8	7.8
Ethyl acetate	2.05 ± 0.37 (1.83)^b^	139.3 ± 19.5	**68.0**
A3	1.14 ± 0.48^a, b^	85.2 ± 6.7	**74.7**
A4	0.32 ± 0.12^a^	151.5 ± 16.2	**473.4**
A5	0.53 ± 0.10^a, b^	989.3 ± 61.3	**1,866.0**
A6	0.51 ± 0.16^a, b^	109.6 ± 20.0	**214.0**
B1	1.79 ± 0.08^b^	142.2 ± 7.3	**79.4**
Luteolin (compound 1)	7.39 ± 0.45^d^	92.9 ± 1.7	**12.6**
Quercetin (positive control)	12.33 ± 0.71^e^	N/A	ND
doxorubicin (positive control)	N/A	9.00 ± 1.28	ND

*N = 2, mean values with a different superscript letters are significantly different at p < 0.05. Values in bold indicate SI > 10. The values in brackets indicate IC_50_ values obtained after log transformation of data which was not normally distributed*.

### Structure Elucidation of Isolated Compounds

#### NMR Results

**Figure 11:**
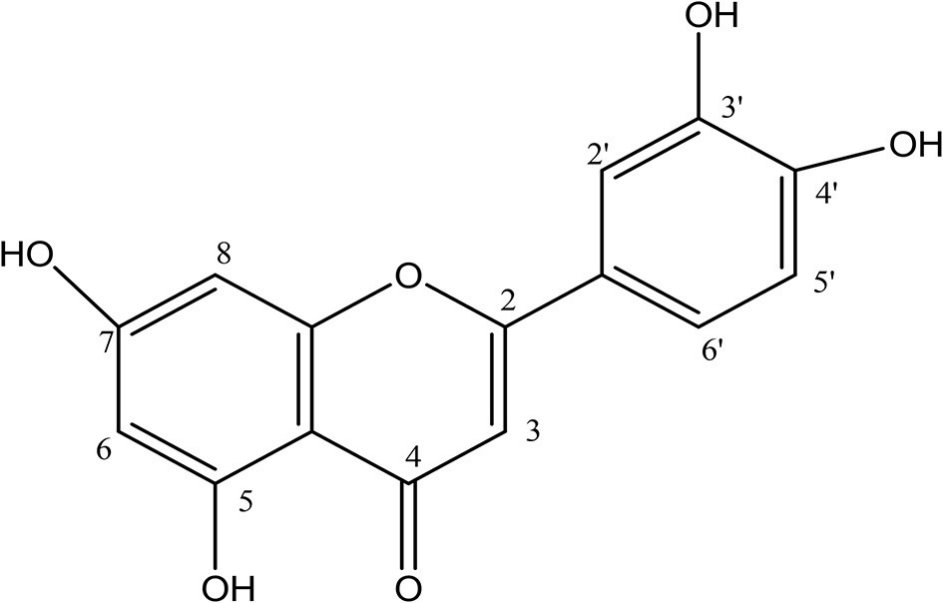
Structure of luteolin.

Analyses of ^1^H and ^13^C NMR revealed that compound 1 was luteolin (Tables 5, 6, Figure 11). The NMR data for compound 1 were similar to that reported for luteolin by da Silva et al. (24). Luteolin appeared as a single yellow band (R_f_ = 0.32) on spraying with vanillin. Compound 2 appeared as a light pinkish single band (R_f_ value = 0.42) whilst compound 3 light greenish single band (R_f_ value = 0.51) after spraying the TLC plate with vanillin (Figure 6A). The structures of compounds 2 and 3 could not be elucidated because they decomposed before analysis. NMR results suggested that they were triterpenoids.

**Table 5:** The ^13^C NMR spectral data of luteolin isolated from *S. singueana*.

**Compound ^**13**^CNMR acetone-d_**6**_, 125 MHz**	**Luteolin ^**13**^CNMR (acetone-d_**6**_, 150 MHz, TMS) (24)**
94.70	94.7 (C-8)
99.70	99.6 (C-6)
103.85	104.2 (C-3)
105.13	105.3 (C-10)
113.88	114.1 (C-2')
116.66	116.6 (C-5')
119.97	120.1 (C-6')
123.09	123.7 (C-1')
146.87	146.6 (C-3')
150.93	150.2 (C-4')
158.75	158.9 (C-9)
163.29	163.3 (C-5)
165.18	164.9 (C-7)
165.24	165.3 (C-2)
182.98	182.9 (C-4)

**Table 6:** The ^1^H NMR spectral data of luteolin isolated from *S. singueana*.

**Compound ^**1**^H NMR (acetone-d_**6**_, 400 MHz)**	**Luteolin ^**1**^H NMR (acetone-d_**6**_, 600 MHz, TMS) (24)**
6.23d	*J* = 2.0	6.25 (1H, d, *J* = 2.1, H-6)
6.52d	*J* = 2.0	6.53 (1H, d, *J* = 2.1,H-8)
6.56s		6.57 (1H, s, H-3)
6.96d	*J* = 8.4	7.00 (1H, d, *J* = 8.4, H-5')
7.44dd	*J* = 8.4; 2.4	7.46 (1H, dd, *J* = 8.4; 2.3, H-6')
7.48d	*J* = 2.4	7.50 (1H, d, *J* = 2.3, H-2')
13.02s		13.00 (1H, s, OH-5)

#### LC/MS Results

The LC/MS results confirmed that compound 1 was luteolin MS (*m/z*) 285.059 (M-H) (Figure 11) with molecular formula (C_15_H_10_ O_6_) and calculated molecular weight of 286.060.

## Discussion

Interest in plant-based feed additives has accelerated following restrictions on the use of antibiotic feed additives in many countries. Plant-derived, or phytogenic, feed additives are being investigated as potential alternatives and to promote their commercial use, they are recommended to have various useful properties to support animal or poultry growth and development. One plant with potential for development into a PFA is *Senna singueana* and this study aimed to evaluate antibacterial, anti-lipoxygenase and antioxidant activity as well as *in vitro* safety of fractions and isolated compounds from the leaf material.

Chromatographic analysis (TLC) using three mobile phase systems did not separate the active compounds of the ethyl acetate fraction into distinct bands, suggesting that the constituent compounds may be closely related. The fact that the active compounds of the dichloromethane and ethyl acetate fractions in the BEA antibacterial bioautography profile did not move from the point of origin shows that the compounds are relatively polar. The BEA solvent system used in the study is a non-polar solvent system.

Column fractionation improved the bioactivity and safety profiles of the *S. singueana* methanol leaf extract, as the fractions were more active than the crude extract. Plant extracts or fractions with MICs of ≤100 μg/ml are considered to have significant activity (25). Fractions A3, A4, and A6 had significant activity against the Gramme-negative bacteria, *E. coli* and *S. Enteritidis* with MICs of <100 μg/ml. Antibacterial activity is a salient feature of PFAs as it is has been postulated that antibiotic feed additives work by modulating gut microflora of animals, thereby preventing sub-clinical infections and also through allowing efficient absorption of nutrients via the thinner intestinal wall associated with antibiotic-fed animals (26, 27). Although fractions A3, A4, and A6 had noteworthy antibacterial activity and were more active than toxic (SI > 1), their safety margins regarding antibacterial activity were low. It is generally considered that biological efficacy is not due to *in vitro* cytotoxicity when SI ≥ 10 (28). Fraction A5 exhibited better safety margins with SI values >3 for all the tested bacterial strains. The SI for fraction A5 regarding *E. coli* was 6.3 which is relatively good. Concerning antibacterial activity, none of the fractions had a therapeutic index of >10, with the most active fractions having selective indexes between 1 and 2.

Antioxidant bioautography revealed that column fractions A2, A3, A4, A5, A6, A7, B1, and B2 had bands of antioxidant activity. Interestingly comparison of the antibacterial and antioxidant bioautography profiles of the main fractions from the first column revealed that most of the compounds which were responsible for antioxidant and antibacterial activity were likely to be the same as they eluted in similar positions. The crude extract of *S. singueana* exhibited powerful antioxidant activity in both the DPPH and ABTS assays. An IC_50_ value of 6.08 μg/ml was recorded with the crude methanol leaf extract in the DPPH assay which was lower than a previously reported IC_50_ value of 20.8 μg/ml obtained from the bark methanol extract of *S. singueana* (8). Quantitative antioxidant analysis also demonstrated that dichloromethane, ethyl acetate and butanol fractions obtained from solvent-solvent partitioning and column fractions A2, A3, A4, A5, A6, A7, B1, B2, and C1 had strong antioxidant activity as there was no significant difference between the IC_50_ values of these fractions and those of the positive controls in both the DPPH and ABTS assays. Antioxidant activity is an important attribute of PFAs. In addition to preventing the oxidative deterioration of feed it also improves the health of the animals. One of the most prevalent poultry diseases, coccidiosis, is associated with oxidative stress caused by the production of free radical oxidative species during the host cellular response to invasion by *Eimeria* species (29). *Eimeria acervulina* oocysts have also been implicated in lipid peroxidation, increased oxidative damage and imbalances in antioxidant status of infected birds caused by disturbing the oxidative balance (30). This implies that PFAs with powerful antioxidant activities can help in the management of this condition. *In vivo* studies have also shown that plant extracts or herbal formulas rich in antioxidants can be effective anticoccidials (31, 32). It is therefore plausible that *S. singueana* fractions can be useful anticoccidials if added to chicken feed although this needs to be verified by carrying out *in vivo* studies.

The SI of the fractions concerning anti-lipoxygenase activity were very good with some having selectivity indexes of >100 with fractions A4 and A5 having very high selective indexes of 473.4 and 1,866.0, respectively. A similar study on the anti-inflammatory activity of crude acetone extract and fractions of *Grewia mollis* reported selective indexes ranging from 1.04 to 54.45 regarding anti-LOX activity (20). Therefore, fractions A3, A4, A5, A6, B1 have the potential to be developed into potent anti-inflammatory agents as they were more anti-inflammatory than toxic. The key enzyme 15-LOX is responsible for the synthesis and release of leukotrienes from poly-unsaturated fatty acids (PUFAs) (20). The *S. singueana* fractions were able to inhibit this enzyme which is involved in the synthesis of pro-inflammatory mediators. The dichloromethane, ethyl acetate and all the evaluated fractions from the first column had better anti-LOX activity than the positive control (quercetin). The IC_50_ values of these fractions were significantly lower than that of quercetin (*p* < 0.5). It has been postulated that antibiotic growth promoters work by permitting growth through an anti-inflammatory role (33). Therefore, anti-inflammatory activity is an important attribute of plant derived products with potential to replace antibiotic growth promoters. Based on their potent antioxidant and anti-lipoxygenase activity, *S. singueana* fractions have potential to be used for the development of poultry phytogenic additives. However, they might need to be blended with other phytochemicals with good antibacterial activity at non-toxic concentrations to produce phytonutrient formulations which can be used effectively as poultry PFAs.

Four tetrahydroanthracene derivatives which showed significant antibacterial and antiplasmodic activity namely singueanol-I and -II, torosachrysone and germichrysone have been previoulsy isolated from the roots of *S. singueana* (34). Other compounds which have been isolated from *S. singueana* include stigmasterol, stigmast-4-en-3- one, stigmast-4,22-dien-3-one, 1-heneicosanol, and hexyl heneicosanoate from the *n*-hexane leaf extract (35). The flavanoid, luteolin has also been isolated from this plant species previously (36). It was found to be responsible for broad anti-ulcer activities of *S. singueana* leaves (36). In the current study, luteolin was also isolated. Luteolin exhibited weak antibacterial activity with MICs of >100 μg/ml being recorded. MICs of >100 μg/ml are not good enough for isolated compounds (21). The results on the antibacterial activity of luteolin were consistent with findings from previous studies which also reported MICs of >100 μg/ml against *E. coli, Staphylococcus* spp., and *Salmonella* spp. (37, 38). However, other researchers have reported significant *in vitro* antibacterial activity of luteolin against *S. aureus, Bacillus subtilis, Listeria monocytogenes, E. coli, Pseudomonas fluorescens*, and *Trueperella pyogenes* (39–41).

Luteolin exhibited strong antioxidant and anti-LOX activity. Previous studies confirmed the strong antioxidant and anti-lipoxygenase activities of luteolin and its mode of action (42–44). The SI of the compound regarding anti-LOX activity was >10. Previous work has also shown that luteolin inhibits cyclooxygenase-II expression (45). The compound can therefore be further investigated as an anti-inflammatory agent. Cycloogenase catalyses the committed step in the synthesis of proinflammatory mediators from arachidonic acid. In addition, luteolin suppressed synthesis of prostaglandin E_2_, a proinflammatory mediator (45). Luteolin also inhibited proinflammatory gene expression in a murine intestinal cell line through the specific modulation of the NF-kB, IRF and Akt signalling pathways (46). Analogous with the *S. singueana* fractions, luteolin has the potential to be included in developing PFA preparations based on its anti-inflammatory activity via different mechanisms. However, it would need to be combined with other compounds with potent antibacterial activity against harmful pathogens, with capacity to act synergistically with it in promoting growth in poultry.

## Conclusion

Column fractions of the ethyl acetate fraction obtained from the crude extract of *Senna singueana* leaves exhibited significant antibacterial, strong antioxidant activity and potent anti-LOX activity and were relatively safe to Vero cells. An active compound, luteolin, which has known biological activities, was isolated together with other compounds. The results of the current study support further investigation of *S. singueana* fractions and luteolin (or its derivatives) for the development of phytonutrient formulations which can be used as alternatives to poultry in-feed antibiotics. *In vivo* work on the formulations should be carried out using broiler chicken models to investigate efficacy as well as safety.

## Data Availability Statement

The raw data supporting the conclusions of this article will be made available by the authors, without undue reservation.

## Author Contributions

PJ conducted the experimental work, analysed the results and wrote the manuscript. FM and GF assisted with bioassays and isolation and structural elucidation of compounds. LM and GM supervised the research and edited the final version. LM provided funding and facilities and submitted the manuscript. All authors revised and edited the manuscript.

## Funding

The National Research Foundation (NRF) of South Africa and TWAS are thanked for providing a Ph.D., scholarship for PJ and research funding to LM (NRF Grant No. 111945).

## Conflict of Interest

The authors declare that the research was conducted in the absence of any commercial or financial relationships that could be construed as a potential conflict of interest.

## Publisher's Note

All claims expressed in this article are solely those of the authors and do not necessarily represent those of their affiliated organizations, or those of the publisher, the editors and the reviewers. Any product that may be evaluated in this article, or claim that may be made by its manufacturer, is not guaranteed or endorsed by the publisher.
